# Exploration of the relationship between laboratory stress parameters and leukocyte coping capacity in ponies exposed to repetitive handling during a research project

**DOI:** 10.1186/s12917-026-05618-1

**Published:** 2026-06-13

**Authors:** Phebe De Heus, Jessica Maute, Nikolaus Huber, Alexander Tichy, Rupert Palme, Eike Steinmann, Asisa Volz, Jessika -M. V. Cavalleri, Dagmar S. Trachsel

**Affiliations:** 1 Clinical Department for Small Animals and Horses, Clinical Centre for Equine Health and Research, University of Veterinary Medicine, Veterinärplatz 1, Vienna, 1210 Austria; 2 Clinical Department for Farm Animals and Food System Science, Center for Food Science and Veterinary Public Health, University of Veterinary Medicine Vienna, Veterinärplatz 1, Vienna, 1210 Austria; 3 Oxford MediStress Ltd, 267 Banbury Rd, Summertown, Oxford, OX2 7HT UK; 4 Department of Biological Sciences and Pathobiology, Bioinformatics and Biostatistics Platform, University of Veterinary Medicine, Veterinärplatz 1, Vienna, 1210 Austria; 5 Department of Biological Sciences and Pathobiology, Unit of Physiology, Pathophysiology and Experimental Endocrinology, University of Veterinary Medicine, Veterinärplatz 1, Vienna, 1210 Austria; 6 Department of Molecular and Medical Virology, Ruhr University Bochum, Universitätsstrasse 150, 44801 Bochum, Germany; 7 Institute of Virology, University of Veterinary Medicine Hannover, Bünteweg 17, 30559 Hannover, Germany

**Keywords:** Cortisol, Neutrophil granulocytes-to-lymphocyte ratio, Neutrophil oxidative burst, PMNL, Polymorphonuclear cells, Equine

## Abstract

**Background:**

Stress is an umbrella term indicating increased allostatic load with the potential to reach a pathological state of the organism. In this context, cortisol concentration and neutrophil granulocytes-to-lymphocyte ratio (NEU: LYM) are expected to increase. On the functional pathway, stress reduces the oxidative burst capacity of neutrophils and therefore, the leucocyte coping capacity (LCC) quantifying this oxygen radical generation of neutrophils. Therefore, we hypothesized that in ponies subjected to repetitive handling, LCC will decrease and will be negatively correlated with cortisol concentration and NEU: LYM ratio. Thus, LCC was measured over 90 min by a portable chemiluminometer in nine university owned ponies involved in a vaccination study that included handling and sampling over 42 days. The area under the curve for LCC (AUC_LCC_) and LCC: NEU (AUC_LCC: NEU_), cortisol concentration measured by a validated EIA and NEU: LYM ratio were compared before and at days 1, 3, 28, 31 and 42 after immunization by linear mixed models. Pearson correlation coefficients were calculated between AUC_LCC_, AUC_LCC: NEU_, cortisol concentration and NEU: LYM ratio.

**Results:**

AUC_LCC_, AUC_LCC: NEU_ and cortisol concentration showed a significant decrease. AUC_LCC_ was moderately positively correlated with NEU: LYM and weakly positively correlated with cortisol concentration, but no correlations were found for AUC_LCC: NEU_. The progressive decrease of AUC_LCC_, AUC_LCC: NEU_, and cortisol concentration might indicate an increasing stress load. However, this was not supported by the time course of NEU: LYM ratio or by the correlations between these parameters.

**Conclusion:**

While the slight decrease in LCC over time partially supported our hypothesis, we found no evidence of correlations among AUC_LCC: NEU_ and the other measured stress parameters. This may indicate that different stress markers reflect distinct endpoints within the physiological pathways of the stress response. Future studies should confirm these preliminary results and also incorporate ethograms to obtain a more comprehensive understanding of the stress response and to further evaluate the value of LCC as a stress marker in horses.

**Supplementary Information:**

The online version contains supplementary material available at 10.1186/s12917-026-05618-1.

## Introduction

Modern equine welfare has evolved beyond merely preventing negative experiences to optimizing positive wellbeing [1]. Therefore, the ability to understand, recognize, and objectively measure the reaction to unpleasant challenges, usually termed “stress”, remains crucial for optimizing horses’ wellbeing. The response to a stressful challenge involves physiological and psychological processes and is mainly mediated through the Hypothalamic-Pituitary-Adrenal (HPA) axis and the autonomic nervous system. Some of the main effector hormones of the HPA axis that can be easily quantified are the adrenocorticotropic hormone (ACTH) and glucocorticoids such as cortisol. Traditionally, markers of stress in equines are cortisol (metabolite) concentrations (measurable in a range of biological substances like blood, faces and saliva) [2–11], behavioural scores and /or ethograms and cardiovascular parameters (i.e. heart rate variability) [5–9, 12–16]. However, these methods often have limitations such as lag time reflecting stress levels, invasiveness, weak correlation between behavioural responses and physiologic markers or susceptibility to external as well as individuals factors [2, 13, 17–28] and coping strategies [29, 30]. Especially the delay between a stressor occurring and the body’s physiological response has led to classify some of the mentioned makers as early markers (i.e. heart rate variability and blood cortisol concentration) or as marker of more long lasting stress (i.e. faecal cortisol) [31]. For other markers (i.e.the neutrophil granulocytes (NEU) to lymphocyte (LYM) ratio the classification is less well defined and will also depend on the stimulus [31].

These limitations have driven an interest in exploring alternative biomarkers. Markers of the immune system are of particular interest as it becomes increasingly evident that there is a multidimensional interaction between the immune system, the endocrine system and the nervous system [32–34]. This complex interaction explains the multiple effects that stress can induce on the immune system through the activated HPA axis and vice-versa. The effect of hormones, especially cortisol, on the immune system can be regulated by direct interaction with receptors on the immune cells [32, 34, 35] or by a modification of inflammatory cytokine production (i.e. INF-g, Il-1, Il-1B, Il-2, Il-6, TNF) [32, 33].

Concerning the immune system, several methods have been used to assess the state of activity of the immune system in the context of stress exposure in horses. These methods include cell counts of leukocytes (LC) and subpopulations, as well as the NEU: LYM ratio to [36–43]. According to this literature and to Gormally et al. [31], the NEU: LYM ratio is considered rater a marker of acute to medium lasting stress. Further, the expression of cytokines, marker molecules or surface receptors [40, 44–46] and functional tests of the immune cells such as NEU [40, 41, 47] or peripheral blood mononuclear cells (PBMC) [44, 46, 48], can give valuable information regarding the effect of stress on the immune system.

One of the functional tests, the leukocyte coping capacity (LCC), is a method used to assess the function of NEU that is part of the innate immune system. This method assesses the capacity of NEU to produce oxygen radicals by measuring the chemiluminescence that occurs when luminol binds with reactive oxygen species released in response to a secondary chemical stimulus [49–52]. The LCC can be performed stall side with a minimal amount of blood, and the scientific literature shows that LCC is significantly reduced affected by pain or stress in general [49, 53–56].

The objective of our pilot study was to obtain initial insights into the assessment of whether the interaction between the immune system and the stress level could be demonstrated by measuring the LCC in ponies that were participating in a study monitoring the immune response during a vaccination protocol. In addition, we aimed to correlate the stress load occurring during handling, as assessed by cortisol concentrations, or NEU: LYM ratio with the LCC measurement.

We hypothesized that the increased stress load induced by handling and sample collection during the vaccination study would increase the stress parameters measured (cortisol levels and NEU: LYM ratios) and decrease the LCC levels.

## Materials and methods

### Animals, holding and medical training

Nine ponies, eight geldings and one mare, were included in the study. The ponies were owned by the University of Veterinary Medicine, Vienna. They belonged to similar pony breeds (7 Shetland-type ponies and 2 American Shetland ponies, other demographic data are reported in Tabel 1).

The ponies were kept at the research facility of the University of Veterinary Medicine, Vienna as a herd in an open stable composed of an outdoor sand paddock with an indoor bedding area containing straw. Hay was available ad libitum from three feed racks, and water was provided ad libitum via an automatic watering system. Any food supplantation or drugs were given. The wellbeing of the ponies was checked twice daily by the stable staff, and the rectal temperature was determined once daily in the morning.

The ponies received medical training (clicker training) twice, one to two days prior to the beginning of the study. This medical training used positive reinforcement. The purpose was to familiarize the ponies with the blood sampling procedure and was intended to reduce the stress load on the ponies during the handling required for the study, as well as to shape their behaviour for overall safety [57]. For medical training and subsequent procedures, the ponies were separated from the herd by leading them to the examination area. The examination area was a ranch rail fenced, straw-free enclosure, within the familiar indoor bedding area, where a separated pony maintained audiovisual contact to the herd. A close cercle of skilled handler restrained the pony with common halter and lead rope.

### Study design

All participating ponies were acquired by the university in early summer 2023 for a larger research project and the ponies received prophylactic vaccinations against endemic infectious pathogens in Austria, including West Nile Virus (WNV). Ethical approval for the vaccination study was obtained from the Ethics Committee of the University of Veterinary Medicine Vienna, and the Austrian Federal Ministry of Education, Science and Research under the Austrian Federal animal use license BMBWF 2022 − 0.118.159, changes documented under 2023 − 0.203.966.

**Fig. 1:**
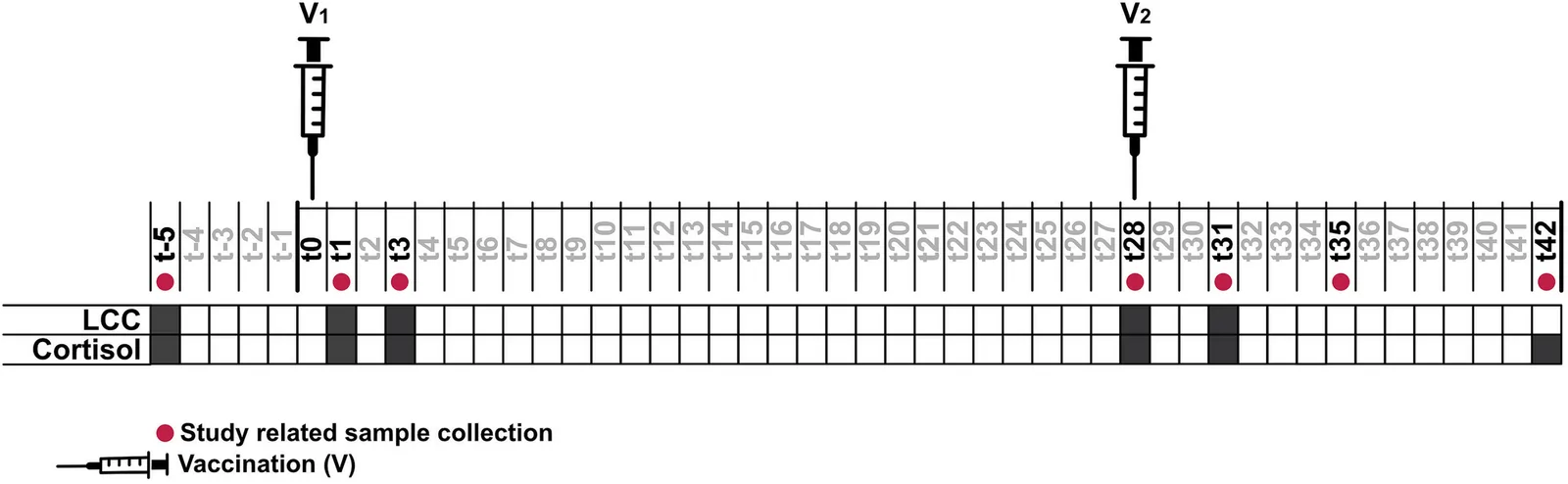
Timeline of the study. Days in black letters represent study related handling in addition to the daily health monitoring of the ponies on each day of the study. Red dots are days with vaccination study related blood/sample collection. Dark grey filled boxes on either the LCC or cortisol line represent analysis of these parameters at the respective day. For the present analyses LCC was measured only on T-5, T1, T3, T28 and T31. Cortisol was measured on T-5, T1, T3, T28, T31 and T42. At T35 samples were taken for the vaccination study purposes, but neither cortisol nor LCC was measured at this time point

The nine ponies were vaccinated with a two-dose vaccination course of a commercially available WNV vaccine (Proteq West Nile™. Boehringer Ingelheim-Vetmedica GmbH, Ingelheim. Germany) according to the manufacturer’s instructions with two intramuscular injections of one ml within a four-week interval. There was no unvaccinated control group. Blood samples for basal values were taken once, five days prior (T-5) to the first vaccination (V1 = T0). Further blood samples for the present study and for monitoring the rise in antibody titres against WNV were taken at one (T1), three (T3), 28 (T28), 31 (T31), and 42 (T42, cortisol only) days after V1, where T28 corresponded to the day of the second vaccination (V2) and T31 to three days after V2 (Fig. 1). An additional blood sample for monitoring the rise in antibody titres against WNV was taken a T21 and T35. Over the entire study period the mean weekly blood volume withdrawn from the ponies ranged from 0.7 to 1.5% of blood volume. This amount was well under the maximal limit of 7.5% of blood volume per week, as outlined by the GV Solas guidelines recommendations for blood sampling in laboratory animals [58].

At each sampling time point for the current study, the ponies were brought to the examination area for clinical examination and sample collection. Ponies were examined and sampled in a fixed order between 9 and 11 o’clock AM and for each sampling day the same sampling protocol was followed. Blood samples were collected by jugular venipuncture with the BD Vacutainer ^®^ -System by an experimented veterinary. During sampling and manipulation, the ponies were given clicks and treats (pieces of carrots or commercial treats from Pferdesporthaus Loesdau GmbH & Co. Bisingen. Germany) as positive reinforcement. The entire manipulation lasted 314 [266–377] seconds (median [IQR]), across all ponies and all time points, including the separation (with audiovisual contact to the rest of the pony herd (supplementary Table T1). The vena jugularis and vaccination injection sites were monitored for complications.

Methodological limitations are that no unvaccinated control group housed under the same environmental conditions was included, and that between T3 and T42 the ponies were exposed to varying degrees of unplanned, non-specific and non-project related stress, such as mild clinical abnormalities, diagnostic handling and manipulations, transportation and at least one non-project related blood sampling by venipuncture (supplementary Table T2 for detailed chronology).

### Blood analysis

The sampled blood was distributed in plain tubes for serum collection, lithium-heparin tubes for plasma collection and in EDTA tubes. The EDTA blood samples were used for haematologic analyses (ProCyte One, IDEXX Vet Med Labor GmbH, Vienna, regularly calibrated and validated according to the quality controls procedures advised by the manufacturer and validated for horses [59]) and the EDTA plasma was used for ACTH measurement with a previously validated method for horses (IMMULITE 2000 ACTH, Siemens Healthcare Diagnostics GmbH, Vienna, Austria) [60, 61]. ACTH was measured to exclude any interference with measured cortisol levels in the ponies remaining in the study at T0.

The lithium-heparin whole blood (10 µl) was taken for LCC measurement. The rest of the sample was centrifuged (3800 g for 5 min) and the plasma separated as soon as possible after sampling and stored at -20 °C until further analyses. Serum was centrifuged after clotting, at 4000 g for 10 min, separated and stored at -80 °C until further analyses. Unfortunately, for one pony no blood sample could be obtained within the defined time frame at T31 due to aversive behaviour, and for two ponies there was insufficient serum available for all analyses (one at T1 and one at T42).

The concentration of serum cortisol was measured by established and validated EIA methods [3, 62, 63]. Biochemical analyses were performed on lithium-heparin plasma with a routine automatised biochemistry analyser (Cobas c501, Roche Diagnostics GmbH, Vienna, Austria) regularly calibrated and validated according to quality controls procedures advised by the manufacturer.

### Measurement of the Leukocyte Coping Capacity (LCC)

The LCC analyses were performed as described by Huber et al. (2019). In brief, LCC was measured every 5 min over 90 min under constant environmental conditions and within 30 min after blood sampling using lithium heparinized whole blood. The LCC was expressed in relative light units (RLU) [53, 64] using a portable chemiluminometer (Leukocyte Coping Capacity™, Oxford MediStress Ltd., Oxford, United Kingdom). This reaction has been shown to be specific for NEU in comparison to PBMCs [52]. The LCC RLU values were then divided by NEU count (LCC: NEU ratio) for each 5 min measurement point to correct for potential mass effect as suggest by the literature [52, 54, 55, 65–67]. Area under de curve (AUC) were calculated for LCC (AUC_LCC_) as well as for the corrected value LCC: NEU (AUC_LCC: NEU_). The utilized protocol stipulated that 90 µl of 10^− 4^ mol/L luminol solution (Sigma-Aldrich, Austria, prepared by dissolving the luminol in dimethyl sulfoxide (DMSO; Merck, Darmstadt, Germany) and further diluting it to the desired concentration with phosphate-buffered saline (PBS 0.01 mol/L, pH 7.4)) was placed into a polystyrol antireflective tube (Greiner Bio-One, Munich, Germany). Then, 10 µl of the lithium heparinized whole blood was added, and the reaction initiated with 10 µl of Phorbol 12-myristate 13-acetate (PMA) of 10^− 5^ mol/L (Merck, Darmstadt, Germany and THP Medical Products, Vienna, Austria) prepared by dissolving it in DMSO (Merck, Darmstadt, Germany) and further diluting it to the desired concentration with PBS 0.01 mol/L, pH 7.4). For each sample, a control tube was run in parallel in which PMA was replaced by PBS 0.01 ml/L, pH 7.4. LCC measurements were only considered valid when control tube showed no RLU activity. Between measurements, the tubes were protected from light and incubated at 37.7 °C in a block heater (MBDB-01 Mini Dry bath, Cleaver scientific, Rugby, UK).

### Statistical analyses

For the statical analyses commercially available designated software was used (GraphPad Prism software 9.5.1. www.graphpad.com. GraphPad Software Boston. MA. USA) and IBM SPSS v29 (IBM Corp., Armonk, NY, USA). GraphPad Prism was also used to create the Figures.

Due to the low number of ponies in the study population and in order to account for possible uneven distribution, the data were described as mean with standard deviation (SD), as median with first and third quantile (Q1. Q3), and as range including maximum (max) and minimum (min) values. The further exploration of the dataset indicated no evidence of violation of assumption for parametric analyses of the data. However, due to the small dataset robust mixed effect models were used to assess the effect of sampling day on the stress parameters or haematological parameters.

Due to the small number of ponies precluding a more explorative approach Pearson correlation coefficients were calculated as a descriptive measure of association between the maximal obtained values of the LCC and LCC: NEU ratio curves and the corresponding AUCs (AUC_LCC_, AUC_LCC: NEU_), as well as between the stress parameters with each other (AUC_LCC_, AUC_LCC: NEU_, plasma cortisol, NEU: LYM ratio). A p-value of < 0.05 was considered statically significant and strength of correlation was interpreted according to. Schober et al. 2018 [68] (0.00–0.10: negligible correlation, 0.10–0.39: weak correlation, 0.40–0.69: moderate correlation, 0.70–0.89: strong correlation, 0.90–1.00: very strong correlation).

The clinical findings at vaccination injection sites or vena jugularis after venipuncture were reported in frequencies but were not included in the analysis.

## Results

### Animals

One pony (pony 7) showed clinical signs of discomfort, hyperthermia, mild hypovolemia at T1 and did not receive the first vaccination as planned. This pony received veterinary attention and oral rehydration, upon which health status was restored, but the pony was subsequently excluded from participation in this study.

At the beginning of the study period (between T1 and T14) all ponies showed transiently elevated liver enzymes (alkaline phosphatase (ALKP) and/or glutamate-dehydrogenase (GLDH) and/or gamma-glutamyl-transferase (GGT), the aspartate-aminotransferase (AST) was also elevated in three of them) [69–71]. Bile acids measured on day T3 in the 8 ponies remaining in the study were within normal range. Based on extensive diagnostics including microbiology sampling, liver biopsy, feed, water and bedding analysis, the cause of the transient liver injury was undetermined. Due to the possibility that the cause was parasitic migration, all the ponies were dewormed at T24 with pyrantel embonate (Banminth, 6.6 mg/kg, Zoetis Zoetis Österreich GmbH, Vienna, Austria). None of the ponies showed clinics sings suggestive of pituitary pars intermedia dysfunction (PPID) and ACTH values (reported in Table 1) were low, with the highest values been 99.00 pg/ml in 8-year-old, female, and therefore not suggestive of PPID in ponies [72, 73].

**Table 1:** Descriptive data of the demographics, hormones of the Hypothalamic-Pituitary-Adrenal (HPA) axis and neutrophil granulocytes-to-lymphocytes ratio (NEU: LYM ratio) of the study population at the various sampling time points

	T-5		T1	T3	T28	T31	T42
Age (years)							
n Mean ± SD Median (Q1; Q3) min-max	9 6.5 ± 5.3 5.0 (2.0; 10.5) 1.0–16.0						
**BW** (kg)							
n Mean ± SD Median (Q1; Q3) min-max	9 135.2 ± 22.2 128.0 (124.0; 146.5) 105.0-184.0						
**ACTH** (pg/ml)							
n Mean ± SD Median (Q1; Q3) min-max	8 24.40 ± 30.35 14.45 (11.30; 18.98) 8.20–99.00						
**Cortisol** (ng/ml)							
n Mean ± SD Median (Q1; Q3) min-max	9 1.69 ± 0.58 1.69 (1.11; 2.06) 0.94–2.75		7 1.71 ± 0.52 1.69 (1.25; 2.16) 1.04–2.50	8 1.81 ± 0.53 1.67 (1.50; 2.14) 1.22–2.90	8 1.26 ± 0.22 1.34 (1.10; 1.41) 0.84–1.54	7 1.34 ± 0.59 1.31 (1.05; 1.50) 0.42–2.42	7 0.96 ± 0.31 0.94 (0.69;1.17) 0.60–1.49
**p value**		**0.008** *	0.954a	0.652a	0.064a	0.236a	**0.007** **a**
**NEU: LYM**							
n Mean ± SD Median (Q1; Q3) min-max	9 0.93 ± 0.35 0.75 (0.65; 1.21) 0.624–1.58		8 1.01 ± 0.42 0.86 (0.61; 1.20) 0.61–1.93	8 0.78 ± 0.24 0.71 (0.55; 0.98) 0.55–1.23	8 0.88 ± 0.32 0.71 (0.62; 1.22) 0.62–1.45	7 0.86 ± 0.18 0.86 (0.67; 0.98) 0.67–1.15	
**p** ** values**		0.701^*^	0.682^a^	0.329^a^	0.790^a^	0.625^a^	

*BW* Body weight, *NEU* neutrophil granulocytes, *LYM* lymphocytes

*p value for the overall fixed effects model; ^a^p value of each time point in comparison to T-5

significant p-values are highlighted in bold

Between T3 and T28, pony 9 developed swollen submandibular lymph nodes, but was otherwise bright, alert and responsive with rectal temperature within normal limits. This pony was subjected to further diagnostic evaluation and as a precaution tested for Streptococcus equi equi (negative on bacteriologic culture and qPCR) from a guttural pouch wash. A tentative diagnosis of lymphadenopathy from transient pharyngitis and change in dentition was made and interpreted as a young age-related process. Pony 9 resumed the study after being found to be healthy by a veterinarian uninvolved in the study.

Two ponies developed a mild and transient swelling, likely a small hematoma, adjacent to the jugular vein after venipuncture (pony 9 at T1 and pony 8 at T35). One of nine ponies (pony 9) developed a mild transient swelling at the injection site after V1 vaccination, and five from nine ponies (pony 1,3,4,6 and 9) developed mild transient swellings at the injection site after V2 vaccination.

### Blood analyses

**Fig. 2:**
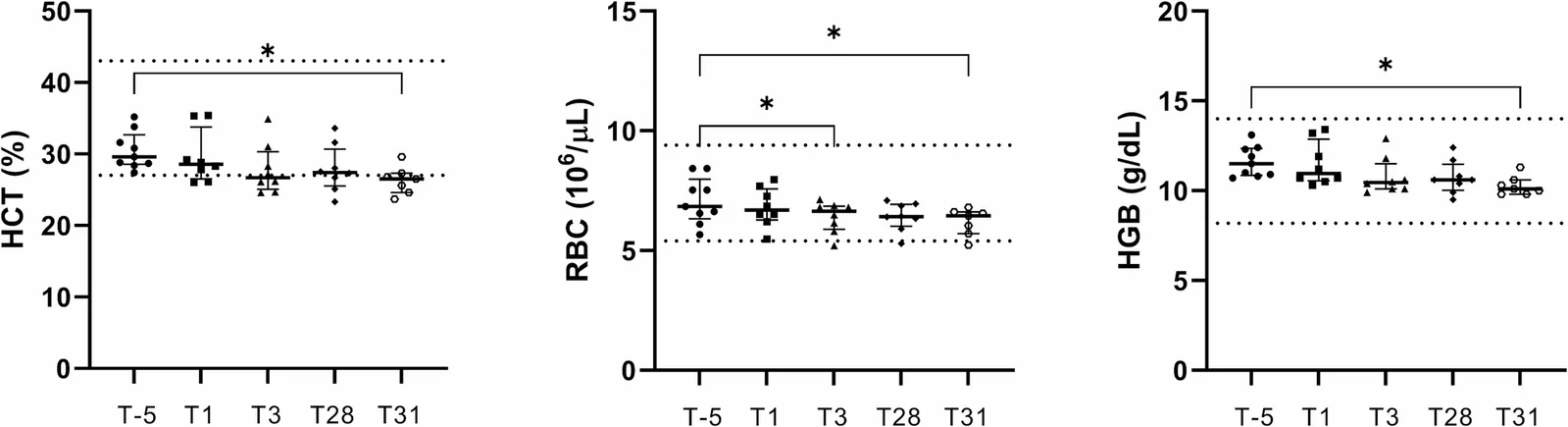
Results of blood haematology at the different timepoints between T-5 and T31 presented as medians and interquartile ranges. The dotted line represents the reference ranges published by Witkowska-Pilaszewicz et al., 2021 [70]. HCT, haematocrit; RBC, number of red blood cells; HGB, haemoglobin concentration; *, *p* < 0,05

The haematocrit (HCT), number of red blood cells (RBC), and haemoglobin (HGB) concentration decreased over time and were statistically different between T-5 and T31 (HCT *p* = 0.012, HGB *p* = 0.010) and between T-5 and T3 and between T-5 and T31 for RBC (*p* = 0.034, *p* = 0.023), but remained within or at the lower end of the reference range according to the most suitable reference range values for our population [70, 71] (Fig. 2).

**Fig. 3:**
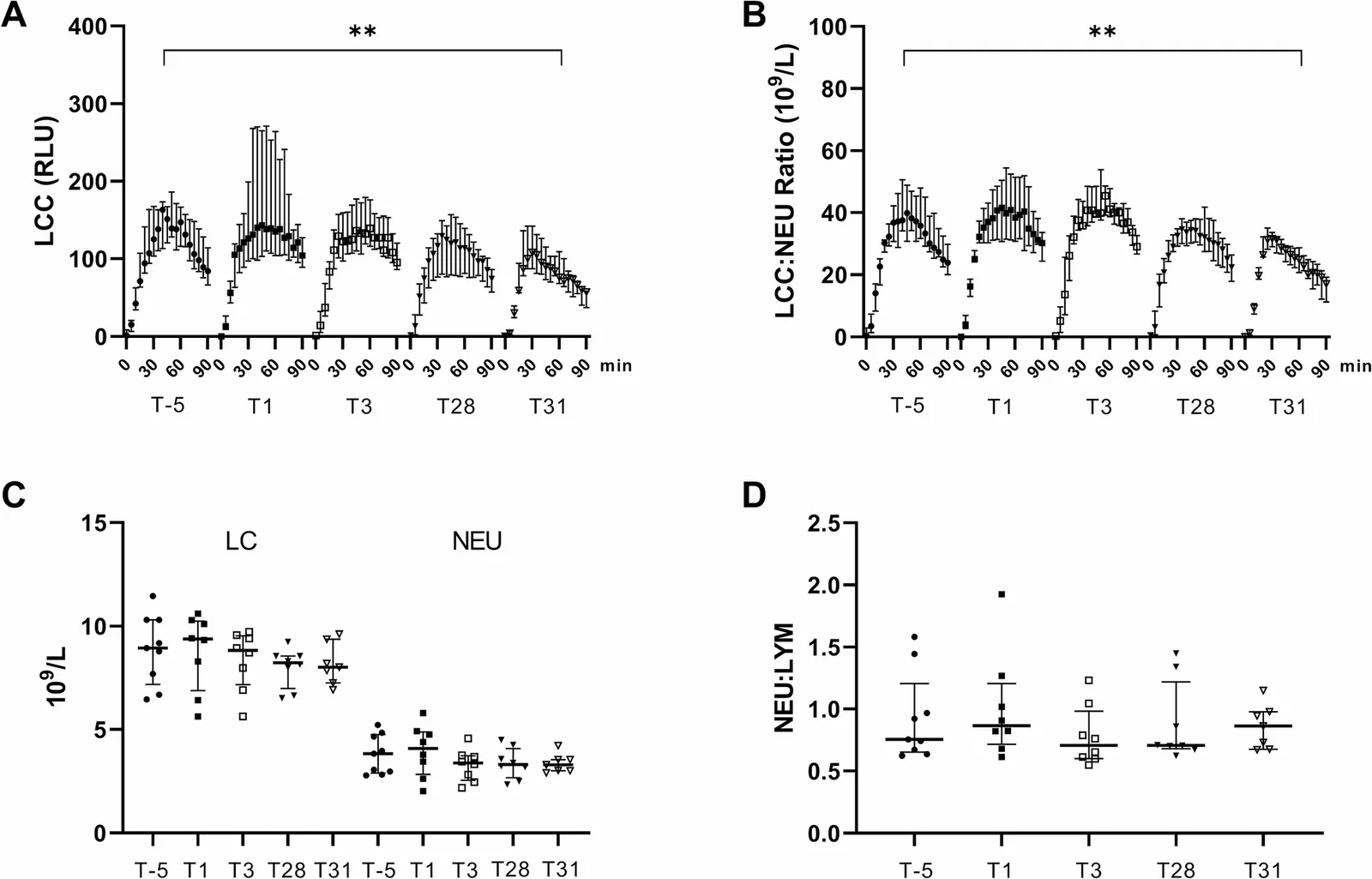
The leukocyte coping capacity (LCC, **A**) expressed as relative light units (RLU) and LCC to neutrophilic granulocyte (NEU) count ratio (LCC: NEU ratio, **B**) is shown at each of the sampling timepoints. Symbols represent median and bars represent Q1-Q3, **, *p* < 0.01. In **C** and **D** the leukocyte (LC) count, the NEU count and the NEU: LYM ratio is shown for the sampling timepoints with symbols for each measurements, median (tick bars) and interquartile ranges (Q1-Q3, thin bars)

The LC (*p* = 0.662) and NEU (*p* = 0.524) counts as well as the NEU: LYM ratio (*p* = 0.701) were constant over the timepoints and were within the reference range according to most suitable reference values for our population [69–71] (Fig. 3C and D; Table 1). Similarly, the monocytes (MON, *p* = 0.068) and LYM (*p* = 0.461) counts were constant over time (supplementary Figure S1).

### LCC measurements

The individual curves for the LCC measurement for each pony are shown in supplementary Figure S1. All LCC measurements were valid except for pony 4 at T1 and T3, and therefore this pony had missing data at T1 and T3.

The correlation between maximum reached value in the LCC (_max_LCC) and LCC: NEU ratio (_max_LCC: NEU) curves and the corresponding AUCs was strong to very strong for all timepoints with the exception of T31 (Table 2). The correlation over all measurement points for _max_LCC values and AUC_LCC_ was also very strong, whereas no correlation could be shown in our data for _max_LCC: NEU and AUC_LCC: NEU_ over all measurement points.

**Table 2:** Pearson correlation coefficients (r) between maximal value of leukocyte coping capacity (LCC) and LCC to neutrophilic granulocyte (NEU) ratio (LCC: NEU) and corresponding areas under the curve (AUC_LCC_ and AUC_LCC: NEU_)

Timepoint	Number of ponies		max LCC		max LCC: NEU	
			r	p-values	r	p-values
T-5	9	AUC_LCC_	**0.991**	**< 0.001**	0.598	0.09
		AUC_LCC: NEU_	0.596	0.09	**1.0**	**< 0.001**
T1	7	AUC_LCC_	**0.975**	**< 0.001**	0.482	0.27
		AUC_LCC: NEU_	0.631	0.13	**0.988**	**< 0.001**
T3	7	AUC_LCC_	**0.888**	**0.008**	0.631	0.13
		AUC_LCC: NEU_	0.719	0.07	**0.879**	**0.009**
T28	8	AUC_LCC_	**0.991**	**< 0.001**	**0.774**	**0.024**
		AUC_LCC: NEU_	0.660	0.07	**0.963**	**< 0.001**
T31	7	AUC_LCC_	0.735	0.06	0.275	0.55
		AUC_LCC: NEU_	0.267	0.56	-0.068	0.88
Overall timepoints	38	AUC_LCC_	**0.905**	**< 0.001**	0.219	0.19
		AUC_LCC: NEU_	**0.669**	**< 0.001**	0.202	0.22

Signigicant r and p-values are highlighted in bold

Based on previous data [64, 65, 67] the strong to very strong correlation at all timepoints except one between AUC_LCC_ and _max_LCC and between AUC_LCC: NEU_ and _max_LCC: NEU was considered sufficient to use AUC_LCC_ and AUC_LCC: NEU_ in the analyses with the mixed models.

The AUC_LCC_ increased slightly from T-5 to T1 and decreased progressively from there until T31. The AUC_LCC: NEU_ increased from T-5 to T1 and to T3 and decrease afterwards until T31. The difference over time for both variables obtained statistical significance between T-5 and T31 (AUC_LCC_ *p* = 0.009 over all time points, *p* = 0.006 T-5 to T31 and AUC_LCC: NEU_ *p* = 0.001 over all timepoints, *p* = 0.009 T-5 to T31, Fig. 3A and B).

### Cortisol

**Fig. 4:**
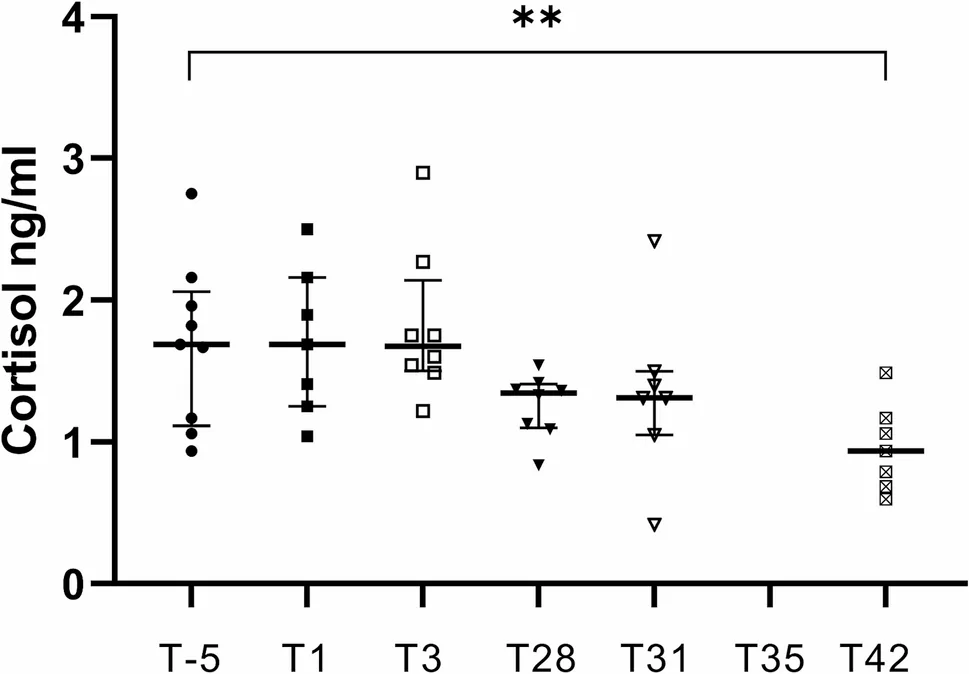
Cortisol concentrations are shown for the sampling timepoints with symbols for each measurement, median (tick bars) and interquartile range (thin bars, Q1-Q3). **, *p* < 0.01

The cortisol concentration increased slightly from T-5 to T1 and T3 and decreased thereafter, to levels even below the basal values of T-5. The difference over time obtained statistical significance between T-5 and T42 (Table 1; Fig. 4).

### Correlations

The correlations between the analyses stress markers are shown in Table 3.

**Table 3:** Pearson correlation coefficient (r) between the determined stress parameters: the leukocyte coping capacity (LCC) expressed as area under the curve (AUC) of LCC and LCC to neutrophilic granulocyte (NEU) ratio (AUC_LCC_ and AUC_LCC: NEU_), the NEU to lymphocyte (LYM) ratio (NEU: LYM), and cortisol

Stress parameter	AUC_LCC: NEU_		NEU: LYM		Cortisol	
	r	p-values	r	p-values	r	p-values
	(n = 38)		(n = 38)		(n=37)	
AUC_LCC_	**0.663**	**< 0.001**	**0.506**	**0.001**	**0.325**	**0.05**
AUC_LCC: NEU_			-0.096	0.565	0.302	0.07
			(*n* = 39)		(*n* = 39)	
NEU: LYM					0.091	0.582

n represents available comparisons varying according to missing data

Significant r and p-values are highlighted in bold

## Discussion

In this pilot study, we aimed at evaluating the stress response induced by study associated handling, manipulations and sampling in ponies participating in a study monitoring the immune response accompanying a two-dose vaccination protocol against WNV over a period up to 42 days. The stress response was evaluated by analysing physiological stress markers such as LCC, cortisol and NEU: LYM ratio over time.

The LCC and LCC: NEU and the corresponding AUCs decreased over time in our study, reaching statistically significant differences between the values at T-5 and those at T31. According to our hypothesis, this result might indicate a progressively increasing stress levels and/or increasing allostatic load over time, rather than an acute stress response due to sampling in this group of ponies. Especially, an acute stress response is not supported by the fact that we found no corresponding changes in NEU: LYM ratio and no increase in cortisol levels. Despite the handling, manipulations and the blood sampling, the measured cortisol concentrations were at the lower range of values published for horses while using the same validated EIA method [3]. The cortisol concentration further decreased over time to reach a statically significant difference between T-5 and T42. The reasons for the overall low levels are unclear. One possible explanation might be that we used serum rather than plasma for cortisol quantification. Differences in matrix composition could theoretically contribute to the discrepancy. However, serum and plasma cortisol concentrations have generally been reported to be equivalent [74], suggesting that the choice of sample type alone is unlikely to fully account for the lower values. Another possible explanation is that ponies may differ from horses in their cortisol concentrations, but such a breed difference has not been reported [75, 76]. However, in ponies, the circannual rhythm appears to be more pronounced, with a visible decrease in cortisol levels during autumn compared with spring [76]. Although this reported reduction was modest, it still suggests lower cortisol concentrations in ponies during the fall season. However, all sampling was performed in the same time frame during the day and over one and half-month period in summer, minimizing both circadian and seasonal influences on cortisol measurements. Finally, all samples were analysed using the same method and at the same time, allowing for direct comparison between animals and time points.

Also, contrary to our initial expectations, we observed a weak to moderate positive correlation between AUC_LCC_ and cortisol concentrations or NEU: LYM ratio. Since we expected the AUC_LCC_ would decrease with increased stress load, the AUC_LCC_ was expected to be negatively correlated with the NEU: LYM ratio and with cortisol concentrations, which increase with stress load [77]. However, these correlations were no longer present, when LCC was corrected for the number of NEU (i.e. AUC_LCC: NEU_). Incongruent findings on the correlations between LCC and cortisol level or NEU: LYM ratio have been published previously. Several studies have reported no correlation between LCC measurements and cortisol concentrations or NEU: LYM ratio [65–67]. In another study, both LCC and AUC_LCC_ increased significantly at 4, 6, and 24 h post-castration in horses, despite concurrent increases in cortisol levels, NEU: LYM ratio, and pain scores, which are typically associated with a stress response [55]. Castration and ensuing surgical wounds, however, could have caused inflammation that activated the NEU [55], influencing their oxidative burst capacity. Similar results have been shown and discussed for calves after castration [56], where LCC and AUC_LCC_ increased 3 days after the procedure. A critical limitation of these previous studies is that LCC was not corrected for the number of NEU, making it difficult to determine whether the observed increase in LCC was due to a true functional change in oxidative burst capacity or simply a higher number of activated NEUs [55, 56]. The authors then further acknowledged that the measured LCC was positively correlated with NEU count and with the ratio AUC/NEU and that despite a change in LCC, this AUC/NEU ratio did not change over time, indicating that the total measured LCC was dependent on the number of cells able to produce an oxidative burst [55, 56]. This highlights the importance of correcting the measured LCC by the number of NEU to account for a mass effect of the cell involved in the overall oxidative bust. In our study we accounted for this by dividing the measured RLU by the number of NEU (i.e. LCC: NEU). In our results the LCC: NEU showed a similar timeline as the LCC, and further, the NEU count was stable over the study period and, even if some of the ponies showed transient low-grade swelling at the injection site or venipuncture site, there was no increase in LC or NEU counts that would have indicated a systemic inflammatory reaction. Therefore, it is not probable that in our study the decreased LCC was related to a change in or particularly, to a decreasing number of NEU. Nevertheless, it seems more reliable to assess the LCC in its relative expression (i.e. LCC: NEU ratio) as reported in our study and in former studies in wildlife [54, 65–67], meaning also that the absence of correlation between AUC_LCC: NEU_ and cortisol concentration or NEU: LYM ratio would be the more reliable finding. In donkeys, vaccination against tetanus and equine influenza has been shown to affect the number of ROS-producing MON, and our vaccination protocol could therefore have influenced MON function as well. However, in our study, MON counts remained constant over time, as did NEU counts. Moreover, MON constitute a small proportion of the LC population compared with NEU, and PBMC function was not affected by PMA in humans [52]. Therefore, we consider it unlikely that a potential reduction in MON function was responsible for the observed changes in LCC measurements.

By measuring LCC every 5 min over 90 min we were able to generate a detailed time profile and calculate the AUC. The AUC in LCC measurements has been used in previous studies [56, 65, 67, 78] and has been shown to correlate very strongly with the maximal measured LCC in deer [65] and in equines [64]. In bears, AUC and maximal LCC values were found to be interchangeable [67]. In our analyses, LCC and AUC exhibited a strong to very strong correlations at most time points, further supporting the use of AUC as a robust summary measure of LCC.

In veterinary medicine, the LCC measurements have mainly been used to assess stress in the context of wildlife manipulation and transport [53, 54, 65–67]. Previous studies compared LCC measurements to various other stress parameters, including the NEU: LYM ratio, a widely used and easily accessible marker of stress. Changes in the NEU: LYM ratio are driven by the physiological responses to stress, whereas elevated catecholamine or cortisol concentrations alter the LC count, leading to an increase in NC counts [36, 37, 39]. In horses, exogenous cortisol administration resulted in an increase in total LC and NC counts at 3 h, 4 h and 8 h post-injection, while LYM count decreased at 4 h, but the NEU: LYM ratio remained increased at 4 h and 8 h post-injection [79]. This response was transient as the cell counts returned to normal values after 24 h. Similarly, following ACTH administration, NEU counts peaked and LYM counts reached their lowest point at 4 h after injection, occurring 2 h after the endogenous cortisol peak [77]. The elevated NEU: LYM ratio persisted for at least 6 h in the study [77]. The time frame and intensity of a stressor appear to be relevant in explaining the changes in the NEU: LYM ratio. Short term physiological stimulation such as a brief bout of exercise or excitement, has been shown to induce lymphocytosis [38, 77]. However, more prolonged or intense stressors, such as exercise to the point of fatigue or endurance races, lead to neutrophilia, which can be accompanied by lymphopenia, starting immediately after exercise and persisting for up to 6 h post-exercise [40, 41, 47]. Similarly, social and environmental stressors can alter leukocyte distribution. Social isolation and exposure to a novel environment has been associated with an increased NEU: LYM ratio [43]. Similarly, transport stress, whether moderate (12 h to 24 h) [42] or prolonged (several days) [46] also induced a shift in the NEU and LYM count with increased NEU and decreased LYM counts, further reinforcing the use of the NEU: LYM ratio as a marker of medium-term stress [31]. However, it is also evident that several external factors may influence this ratio. A study comparing the NEU: LYM ratio to LCC measurement in stallions after castration measured an increased NEU: LYM ratio 4 h, 6 h and 24 h after the surgery [55] found that the NEU: LYM ratio was positively correlated with LCC and pain scores [55]. However, it is important to consider that surgical inflammation could have impacted the NEU and LYM counts as well as the LCC in this previous study [55].

The NEU: LYM ratio measured in our study was consistent with values previously reported for ponies [70, 80] and remained stable over time. Therefore, the stability of the time profile of this parameter does not support the hypothesis that the ponies experienced an increasing or chronic stress load despite exposure to multiple potential stressors. Furthermore, the NEU: LYM ratio showed a weak positive correlation to AUC_LCC_ but not to AUC_LCC: NEU_. This aligns with findings from studies assessing stress response in wildlife during capture or transport where no correlation between NEU: LYM ratio and LCC was observed [65–67]. Interestingly, similar to our results, these previous studies [65–67] also found no correlation between cortisol concentrations and NEU: LYM ratio. Taken together, this lack of correlation between different stress markers suggests that each parameter may reflect distinct aspects of the physiological stress response.

Measurement of cortisol (or its metabolites), in blood, saliva or faeces has been widely used to assess the HPA axis of the stress response in wildlife as well as in horses [2–10, 65–67]. Nonetheless, there are ongoing debates as to whether cortisol measurements can distinguish between short lasting states of arousal and physical stimulation compared to long-lasting stress or between physical stress compared to psychological or social stress [2, 17, 28, 30, 31, 81]. Especially in comparison with increasing cortisol concentrations after short lasting stimuli as exercise, transport or manipulation [2, 5–9, 40, 77], lower cortisol concentrations have been associated with longer lasting exposure to stress and in welfare compromising situations [30, 81, 82]. The interpretation of cortisol concentrations is further complicated by the fact that blood cortisol is influenced by many individual factors, such as age, breed or coping strategy [29, 30] as well as environmental factors, including the nature and valence of the stressor [17, 20–26]. Additionally, cortisol concentration follows a circadian rhythm peaking in the morning [83–85], which makes consistent sampling times essential for reliable comparisons. Ideally also the season should be taken into consideration as slightly higher values are measured in spring time [85]. In our study the blood samples were collected between 9:00 and 11:00 AM over and one and a half-month period in summer, minimizing both circadian and seasonal influences on cortisol measurements. Overall the measured cortisol concentrations were at the lower range of values published for horses using the same validated EIA [3] and the cortisol concentration decreased over the study period. Therefore, this pattern does not support an acute stress response caused by the handling before or during blood sampling. However, low cortisol concentrations have been reported in horses with more chronic exposures to stress [30, 81] and we cannot completely exclude the possibility that the separation of the herd, manipulations, handling, but also the transport and the repetitive blood sampling induced a chronic state of stress in the ponies, despite the implemented clicker training, as could have been supported by the results of LCC measurements. Similarly, the statistically significant decrease over time in HCT, RBC, and HGB could possibly have added to the stress load. However, the values remained within the suitable reference range values for our population [70, 71] and therefore the results of the haematologic analyses was not indicative of a clinically relevant change in oxygen carrier capacity, that could have increased a chronic sate of stress in the ponies.

Further, even if we sampled all sample for cortisol measurement within a strict time frame to account for circadian rhythms and that the ponies had audiovisual contact to the others over the whole sampling procedure, we cannot completely exclude an effect of the sampling order in cortisol measurement. Unfortunately, faecal samples considered more reliable to assess a more long-lasting stress load were not taken despite the fact that they are non-invasive and are not influenced by handling [11, 21, 30, 62, 81].

Due to logistical constraint and limited staffing capacity, it was not possible to include LCC measurements and NEU: LYM ratio beyond T31, and therefore the comparison of these stress parameters could only be done until T31. However, the inclusion of cortisol measurement at T42 is interesting as it supports well the overall pictures of a rather long-term effect in our pilot study. However, assessing behaviour by an ethogram or behaviour scores would have added valuable information regarding the stress load of the ponies during the study period. In particular, this information would have been able to contribute to the assessment of the stress load during handling and/or between study-related manipulations when the ponies were kept in an open stable with permanent access to an outdoor sand paddock with no control over the load of environmental stress factors such as insects, or excessive heat. Also, inclusion of a control group living at the same location with no vaccination or study-related manipulation would have provided information on the environmental stress load during the study period. Therefore, it is not possible to assess to what extend other environmental factors might have influenced our measurements. Additionally, it was not possible to control for all external events that the ponies were exposed to throughout the study period, nor could we ensure that the same close group of individuals consistently handled the ponies during the blood sampling. While Ijichi et al. reported that the presence of unfamiliar people does not significantly influence stress reactions in horses [86], we cannot completely rule out the possibility that handler variability contributed to individual differences in stress load.

To gain a more comprehensive understanding of stress responses, future studies should incorporate validated ethograms or behavioural scoring systems for comparison with the LCC values. Integrating behavioural data alongside LCC measurements in ponies would contribute to the assessment of long-lasting or psychological stress, as has been described in water voles and non-human primates in various housing systems [78, 87] or bears and water voles in difference social group settings [67, 88].

Finally, due to the small number of available ponies the results should be considered as a pilot study. Especially, due to the limited number of ponies and the subsequent small dataset we were constrained to use more descriptive approaches that might not be the optimal. Especially, we choose to use a Pearson correlation ignoring independency as more complex analyses would have been unreliable in such context. Also, the absence of correlation between _max_LCC and _max_LCC: NEU to the corresponding AUC at one time point is most probably due to unstable statistic with the present small data set. While our findings provide valuable insights into the physiological stress response in ponies, these limitations highlight the need for a more comprehensive approach in future research to strengthen conclusions regarding the role of LCC in assessing long-term stress.

## Conclusion

In conclusion, in this pilot study, LCC and LCC: NEU ratios were measured and compared to NEU: LYM ratios and cortisol concentrations in nine ponies over a 31-day period, during which the animals were exposed to handling, manipulations, vaccination, and repeated sample collection.

Our findings, in line with previous literature [64, 65, 67]. confirmed that the AUC for LCC measurements over 90 min, correlated strongly with maximal LCC values (_max_LCC and _max_LCC: NEU). This supports the use of AUC as a reliable measure of LCC in ponies.

Since the LCC assesses the oxidative burst capacity of cells involved in the immune response, it is crucial to consider the total number of available cells in this reaction. In other words, the LCC should be corrected for a possible mass effect of increased or decreased NEU counts.

After applying this correction, the AUC (i.e. AUC_LCC: NEU_) showed no correlation with cortisol concentration or NEU: LYM ratio in ponies, which is consistent with findings in other species [65–67]. These results emphasize that various stress markers reflect different and distinct endpoints on the physiological pathways within the stress response, rather than providing a single, unified measure of stress.

The progressive decrease of AUC_LCC_, and AUC_LCC: NEU_ and cortisol concentration observed in this pilot study may suggest increasing stress or allostatic load over time. However, this interpretation is not supported by the time course, the NEU: LYM ratios, or the correlations between these stress markers.

To improve the validity of LCC as a stress marker, additional immunological or hormonal stress markers as well as behavioural assessment during the exposure to stress should be included in the study design and careful standardization of the stress stimulus is essential in order to ensure comparability of findings.

## Supplementary Information

Supplementary Material 1: Appendix A supplementary Figure S1: Differential cell count.

Supplementary Material 2: Appendix A supplementary Figure S2: Individual LCC curves.

Supplementary Material 3: Appendix A supplementary Table 1: Time recordings.

Supplementary Material 4: Appendix A supplementary Table 2: Chronology.

## Data Availability

The datasets used and/or analyzed during the current study are available from the corresponding author on reasonable request.
