# Supplementary material for: Exploration of the relationship between laboratory stress parameters and leukocyte coping capacity in ponies exposed to repetitive handling during a research project
Source: BMC Vet Res. 2026 Jun 13;22:483. doi: 10.1186/s12917-026-05618-1 (PMC13495423; doi:10.1186/s12917-026-05618-1)
Supplement: Supplementary file 1 [file 12917_2026_5618_MOESM1_ESM.docx]

**Supplementary figure S1:** Differential cell counts for the nine ponies at the 5 measurement timepoints presented as medians and interquartile ranges. The dotted line represents the reference ranges published by Köller et al. 2014 [69]. LC, leukocyte; LYM, lymphocytes; MON, Monocytes; NEU, Neutrophilic granulocytes; EOS, eosinophilic granulocytes; BASO, basophilic granulocytes.
