# Supplementary material for: Exploration of the relationship between laboratory stress parameters and leukocyte coping capacity in ponies exposed to repetitive handling during a research project
Source: BMC Vet Res. 2026 Jun 13;22:483. doi: 10.1186/s12917-026-05618-1 (PMC13495423; doi:10.1186/s12917-026-05618-1)
Supplement: Supplementary file 2 [file 12917_2026_5618_MOESM2_ESM.docx]

**Supplementary figure S1:** LCC:NEU measured over 90 min for each pony at different sampling time points. 4 ponies showed a decrease in the LCC:NEU curve over the 5 sampling timepoint (A), 2 ponies show almost constant LCC:NEU curves (B) and for 3 ponies the sampling was incomplete (C).

**A**

**C**

**B**
