# Supplementary material for: Exploration of the relationship between laboratory stress parameters and leukocyte coping capacity in ponies exposed to repetitive handling during a research project
Source: BMC Vet Res. 2026 Jun 13;22:483. doi: 10.1186/s12917-026-05618-1 (PMC13495423; doi:10.1186/s12917-026-05618-1)
Supplement: Supplementary file 3 [file 12917_2026_5618_MOESM3_ESM.docx]

**Supplementary table T1: Time recordings for handing and blood sampling**

|  | **Separation time** | | | | | | |  |
| --- | --- | --- | --- | --- | --- | --- | --- | --- |
|  | **Time point in the study (days after vaccination V1)** | | | | | | |  |
| **Pony nr** | **T0** | **T1** | **T3** | **T28** | **T31** | **T35** | **T42** | **Median/pony** |
| **1** | 343 | 301 | 341 | 252 | 298 | 241 | 222 | 298 |
| **2** | 298 | 324 | 403 | 288 | 281 | 231 | 354 | 298 |
| **3** | 424 | 301 | 312 | 341 | 340 | 279 | 246 | 312 |
| **4** | 347 | 303 | 608 | 376 | 294 | 260 | 259 | 303 |
| **5** | 294 | 378 | 269 | 257 | 323 | 497 | 497 | 323 |
| **6** | 319 | 315 | 143 | 279 | 229 | 220 | 316 | 279 |
| **8** | 351 | 449 | 519 | 968 | 667 | 899 | 553 | 553 |
| **9** | 363 | 313 | 188 | 434 | 245 | 245 | 654 | 313 |
| **Median/time point** | 345 | 314 | 327 | 314 | 296 | 252 | 335 |  |
|  |  |  |  |  |  |  |  |  |

Time recordings of sampling in seconds. Time recordings started after pony was brought into the examination area and after a clinical examination.
