# Supplementary material for: Exploration of the relationship between laboratory stress parameters and leukocyte coping capacity in ponies exposed to repetitive handling during a research project
Source: BMC Vet Res. 2026 Jun 13;22:483. doi: 10.1186/s12917-026-05618-1 (PMC13495423; doi:10.1186/s12917-026-05618-1)
Supplement: Supplementary file 4 [file 12917_2026_5618_MOESM4_ESM.docx]

**Supplementary table T2: Chronology of the events occurring during the study period**

| **Date** | **Time points** | | **Ponys (Po)** | **Events** | **Clinical exam** | **Laboratory analyses** | **Medication** |
| --- | --- | --- | --- | --- | --- | --- | --- |
| 19.06.2023 | T-7 |  | Po1, Po2, Po3, Po4, Po5 | Medical training day 1 |  |  |  |
|  |  | T-8 | Po6, Po7, Po8, Po9 | Medical training day 1 |  |  |  |
| 20.06.2023 | T-6 |  | Po1, Po2, Po3, Po4, Po5 | Medical training day 2 |  |  |  |
|  |  | T-7 | Po6, Po7, Po8, Po9 | Medical training day 2 |  |  |  |
| 21.06.2023 | T-5 |  | Po1, Po2, Po3, Po4, Po5 | Blood sampling for the present study and for monitoring the rise in antibody titres against the WNV | normal | Full haematological and biochemical analyses for health check prior of the study begin: all parameters including liver enzymes (GLDH, GGT, ALKP, AST) within normal limits (wnl) LCC  Cortisol  NEU:LYM ratio |  |
| 22.06.2023 |  | T-5 | Po6, Po7, Po8, Po9 | Blood sampling for the present study and for monitoring the rise in antibody titres against the WNV | normal | Full haematological and biochemical analyses for health check prior of the study begin: all parameters including liver enzymes (GLDH, GGT, ALKP, AST) within normal limits (wnl) LCC  Cortisol  NEU:LYM ratio |  |
| 23.-25.06.2023 |  | |  | uneventful |  |  |  |
| 26.06.2023 |  | T-1 | Po7 | excluded from the study, not vaccinated Blood sampling for diagnostics | Hyperthermia, discomfort and mild hypovolemia | Liver enzymes (GLDH and GGT) increased | Voluntary oral hydration with tap water |
|  | T0 |  | Po1, Po2, Po3, Po4, Po5 | Vaccination V1 | normal |  | Proteq West Nile™ (Boehringer Ingelheim Vetmedica GmbH, Ingelheim, Germany) |
| 27.06.2023 |  | T0 | Po6, Po8, Po9 | Vaccination V1 | normal |  | Proteq West Nile™ (Boehringer Ingelheim Vetmedica GmbH, Ingelheim, Germany) |
|  | T1 |  | Po1, Po2, Po3, Po4, Po5 | Blood sampling for the present study and for monitoring the rise in antibody titres against the WNV. | normal | LCC  Cortisol  NEU:LYM ratio |  |
| 28.06.2023 |  | T1 | Po6, Po8, Po9 | Blood sampling for the present study and for monitoring the rise in antibody titres against the WNV. | normal, except  Po9 with transient swelling adjacent to the jugular vein and transient swelling at the injection site after V1 | LCC  Cortisol  NEU:LYM ratio |  |
| 29.06.2023 |  | T2 | Po6, Po8, Po9 | Blood for liver diagnostics (after having obtained results from Po7) | normal | Liver enzymes (GLDH and/or GGT, and/or ALKP) increased, bile acids wnl |  |
|  | T3 |  | Po1, Po2, Po3, Po4, Po5 | Blood samples for the present study, for monitoring the rise in antibody titres against the WNV and blood for liver diagnostics (after having obtained results from Po7 | normal | Liver enzymes (GLDH and/or GGT) increased, bile acids wnl  LCC  Cortisol  NEU:LYM ratio |  |
| 30.06.2023 |  | T3 | Po6, Po8, Po9 | Blood samples for the present study and for monitoring the rise in antibody titres against the WNV. |  | LCC  Cortisol  NEU:LYM ratio |  |
| 01.-02.07.2023 |  | |  | uneventful |  |  |  |
| 03.07.2023 |  | T6 | Po6, Po8, Po9 | Ultrasound liver (for furthers liver diagnostics in purpose of the larger planned study): normal | normal |  |  |
|  | T7 |  | Po1, Po2, Po3, Po4, Po5 | Ultrasound liver (for furthers liver diagnostics in purpose of the larger planned study): normal | normal |  |  |
|  |  |  | Po1, Po2, Po4 | Blood sampling for Hepaciviral diagnostic (in purpose of the larger planned study) |  | qPCR form serum negative for hepacivirus |  |
| 04.-09.07.2023 |  | |  | uneventful |  |  |  |
| 10.07.2023 | T14 |  | Po1, Po2, Po3, Po4, Po5 | Blood sampling for control of liver enzymes | normal | Liver enzymes: GLDH wnl/ GGT wnl, except for two ponies: |  |
| 11.07.2023 |  | T14 | Po6, Po8, Po9 | Blood sampling for control of liver enzymes | normal, except Po9 with enlarged submandibular lymph nodes (Lnn_subm_), not painful otherwise clinical exam normal | Liver enzymes: GLDH wnl, GGT increased |  |
|  |  | T14 | Po9 | Transport to the equine hospital for diagnostics of lymphadenopathy without fever |  |  |  |
|  | T15 |  | Po1, Po2, Po4 | Transport to the equine hospital for diagnostics of increased liver enzymes | normal |  |  |
| 12.07.2023 |  | T15 | Po9 | Sedation and endoscopy of upper airways incl. Lavage of guttural pouch for strangles diagnostics | Lnn_subm_ still enlarge and not painful | qPCR and bacterial culture negative for streptococcus equi equi. | Detomidine 0.01 mg/kg IV (Domidine, Dechra Veterinary Products GmbH, Dornbin,, austria),  Butorphanol 0.01 mg/kg IV (Butomidor, VetViva Richter GmbH, Wels, Austria |
| 13.07.2023 | T17 |  | Po1, Po2, Po4 | Sedation and liver biopsy (for furthers liver diagnostics in purpose of the larger planned study) |  | Bacteriological exam of the liver biopsies negatives in sampled all ponies  Histology:  Po1: mild and unspecific degeneration of hepatocytes (probably due to fat infiltration), biopsy score by Durham et al. 2003: 1  Po2: mild lymphoplasmacytic and eosinphilic portal hepatitis / pericholangitis, mild unspecific, ,centrolobular vacuolar degeneration of hepatocytes (probably due to fat infiltration), biopsy score by Durham et al. 2003: 1-2  Po4: mild lymphoplasmacytic portal hepatitis / pericholangitis, unspecific, biopsy score by Durham et al. 2003: 1-2: | Detomidine 0.01 mg/kg IV (Domidine, Dechra Veterinary Products GmbH, Dornbin,, austria),  Butorphanol 0.01 mg/kg IV (Butomidor, VetViva Richter GmbH, Wels, Austria Flunixine meglumine 1.1 mg/kg IV (Niglumine, Vana GmbH, Vienna, Austria) |
| 14.-16.07.2023 |  | |  | uneventful |  |  |  |
| 17.07.2023 | T21 |  | Po3, Po5 | Blood samples for monitoring the rise in antibody titres against the WNV. |  |  |  |
| 18.07.2023 |  | T21 | Po9 | Sedation and dental examination | Lymphadenopathy probably due to change in dentition, Lnn_subm_ only slightly enlarged still not painful |  | Detomidine 0.01 mg/kg IV (Domidine, Dechra Veterinary Products GmbH, Dornbin,, austria),  Butorphanol 0.01 mg/kg IV (Butomidor, VetViva Richter GmbH, Wels, Austria |
|  |  | T21 | Po6, Po8 | Blood samples for monitoring the rise in antibody titres against the WNV. |  |  |  |
| 19.07.2023 |  | |  | uneventful |  |  |  |
| 20.07.2023 | T24 |  | Po1, Po2, Po3, Po4, Po5 | Deworming (following identification of Oxiuris equi infection while taking rectal temperatures) | normal | Microscopy of adhesive tape positive for oxyuris eggs | Pyrantel 6,6 mg/kg PO (Banminth, Zoetis Österreich GmbH, Vienna, Austria) |
|  |  | T23 | Po6, Po8, Po9 | Deworming (following identification of Oxiuris equi infection while taking rectal temperatures | normal | Microscopy of adhesive tape positive for oxyuris eggs | Pyrantel 6,6 mg/kg PO (Banminth, Zoetis Österreich GmbH, Vienna, Austria) |
| 21.07.2023 | T25 |  | Po1, Po2, Po4 | Transport back to research facility | normal |  |  |
|  |  | T24 | Po9 | Transport back to research facility | normal |  |  |
| 22.-23.07.2023 |  | |  | uneventful |  |  |  |
| 24.07.2023 | T28 |  | Po1, Po2, Po3, Po4, Po5 | Vaccination V2  Blood sampling for the present study and for monitoring the rise in antibody titres against the WNV. | normal | LCC  Cortisol  NEU:LYM ratio | Proteq West Nile™ (Boehringer Ingelheim Vetmedica GmbH, Ingelheim, Germany) |
| 25.07.2023 |  | T28 | Po6, Po8, Po9 | Vaccination V2  Blood sampling for the present study and for monitoring the rise in antibody titres against the WNV. | normal | LCC  Cortisol  NEU:LYM ratio | Proteq West Nile™ (Boehringer Ingelheim Vetmedica GmbH, Ingelheim, Germany) |
| 26.07.2023 |  | |  | uneventful | Po1, Po 3, Po 4, Po 6 and Po 9 developed mild transient swellings at the injection site after V2 |  |  |
| 27.07.2023 | T31 |  | Po1, Po2, Po3, Po4, Po5 | Blood sampling for the present study and for monitoring the rise in antibody titres against the WNV. | normal | LCC  Cortisol  NEU:LYM ratio |  |
| 28.07.2023 |  | T31 | Po6, Po8, Po9 | Blood sampling for the present study and for monitoring the rise in antibody titres against the WNV. | normal | LCC  Cortisol  NEU:LYM ratio |  |
| 29.-30.07.2023 |  | |  | uneventful |  |  |  |
| 31.07.2023 | T35 |  | Po1, Po2, Po3, Po4, Po5 | Blood sampling for monitoring the rise in antibody titres against the WNV. | normal | No sampling for the present study due to limited staffing capacity |  |
| 01.08.2023 |  | T35 | Po6, Po8, Po9 | Blood sampling for monitoring the rise in antibody titres against the WNV. | Normal, except Po8 with transient swelling, adjacent to the jugular vein | No sampling for the present study due to limited staffing capacity |  |
| 02.-06.08.2023 |  | |  | uneventful |  |  |  |
| 07.08.2023 | T42 |  | Po1, Po2, Po3, Po4, Po5 | Blood sampling for cortisol and for monitoring the rise in antibody titres against the WNV. | normal | Cortisol  No sampling for the other stress parameters due to limited staffing capacity |  |
|  |  | T41 | Po6, Po8, Po9 | Blood sampling for cortisol and for monitoring the rise in antibody titres against the WNV | normal | Cortisol  No sampling for the other stress parameters due to limited staffing capacity |  |

ALKP, alkaline phosphatase; AST, aspartate-aminotransferase; GGT, gamma-glutamyl-transferase; GLDH, Glutamatdehydrogenase; LCC, Leukocyte Coping Capacity, Lnn_subm_, submandibular lymph nodes; NEU:LYM ratio, neutrophil granulocytes (NEU) to lymphocyte (LYM) ratio; Po, Ponys; wnl, within normal limits; WNV, West Nile Virus.

Durham AE, Smitht KC, Newton JR, Hillyer MH, Hillyer LL, Smith MRW, Marr CM: Development and application of a scoring system for prognostic evaluation of equine liver biopsies. Equine Vet J 2003, 35(6):534-40. doi: 10.2746/042516403775467171
